# *N*′-[(*E*)-2-Chloro­benzyl­idene]-2-[(1,3,4-thia­diazol-2-yl)sulfan­yl]acetohydrazide

**DOI:** 10.1107/S1600536811035549

**Published:** 2011-09-14

**Authors:** S. Mohan, S. Ananthan, P. Ramesh, D. Saravanan, M. N. Ponnuswamy

**Affiliations:** a Research and Development Centre, Orchid Chemicals and Pharmaceuticals Ltd, Chennai 600 119, India; b Department of Chemistry, Presidency College (Autonomous), Chennai 600 005, India; c Centre of Advanced Study in Crystallography and Biophysics, University of Madras, Guindy Campus, Chennai 600 025, India; d Department of Chemistry, National College, Tiruchirappali 620 001, India

## Abstract

In the title compound, C_11_H_9_ClN_4_OS_2_, the thia­diazole and chloro­phenyl rings are oriented at an angle of 43.1 (1)°. The sum of the bond angles around the amide N atom (359.8°) of the acetohydrazide group is in accordance with a model of *sp^2^* hybridization. In the crystal, inversion dimers linked by pairs of N—H⋯O hydrogen bonds generate *R*_2_^2^(8) loops. Weak C—H⋯π inter­actions also occur.

## Related literature

For related literature on the biological activities of 1,3,4-thiadia­zole derivatives, see: Alireza *et al.* (2005 ▶); Matysiak & Opolski (2006 ▶); Wang *et al.* (1999 ▶). For hydrogen-bond motifs, see: Bernstein *et al.* (1995 ▶).
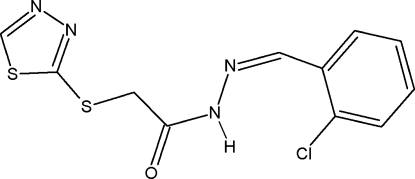


## Experimental

### 

#### Crystal data

C_11_H_9_ClN_4_OS_2_
*M* *_r_* = 312.79
Triclinic, 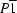

*a* = 7.551 (5) Å
*b* = 8.743 (3) Å
*c* = 11.269 (5) Å
α = 69.295 (5)°
β = 87.493 (4)°
γ = 78.892 (5)°
*V* = 682.6 (6) Å^3^
*Z* = 2
Mo *K*α radiation
μ = 0.58 mm^−1^
*T* = 293 K
0.20 × 0.17 × 0.16 mm

#### Data collection

Bruker SMART APEXII area-detector diffractometer
Absorption correction: multi-scan (*SADABS*; Bruker, 2008 ▶) *T*_min_ = 0.890, *T*_max_ = 0.911
12836 measured reflections
3446 independent reflections
2858 reflections with *I* > 2σ(*I*)
*R*_int_ = 0.026

#### Refinement

*R*[*F* ^2^ > 2σ(*F* ^2^)] = 0.043
*wR*(*F* ^2^) = 0.121
*S* = 1.05
3446 reflections
176 parameters
H atoms treated by a mixture of independent and constrained refinement
Δρ_max_ = 0.81 e Å^−3^
Δρ_min_ = −0.70 e Å^−3^

### 

Data collection: *APEX2* (Bruker, 2008 ▶); cell refinement: *APEX2*; data reduction: *SAINT* (Bruker, 2008 ▶); program(s) used to solve structure: *SHELXS97* (Sheldrick, 2008 ▶); program(s) used to refine structure: *SHELXL97* (Sheldrick, 2008 ▶); molecular graphics: *ORTEP-3* (Farrugia, 1997 ▶); software used to prepare material for publication: *SHELXL97* and *PLATON* (Spek, 2009 ▶).

## Supplementary Material

Crystal structure: contains datablock(s) global, I. DOI: 10.1107/S1600536811035549/bt5593sup1.cif

Structure factors: contains datablock(s) I. DOI: 10.1107/S1600536811035549/bt5593Isup2.hkl

Supplementary material file. DOI: 10.1107/S1600536811035549/bt5593Isup3.cml

Additional supplementary materials:  crystallographic information; 3D view; checkCIF report

## Figures and Tables

**Table 1:** Hydrogen-bond geometry (Å, °) *Cg*1 is the centroid of the S1/C1/N2/N3/C4 ring.

*D*—H⋯*A*	*D*—H	H⋯*A*	*D*⋯*A*	*D*—H⋯*A*
N7—H7⋯O1^i^	0.91 (3)	1.93 (3)	2.845 (3)	175 (2)
C5—H5*A*⋯*Cg*1^ii^	0.97	2.95	3.896(3)	165

Symmetry codes: (i) 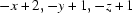; (ii) 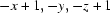.

**Fig. 1.**
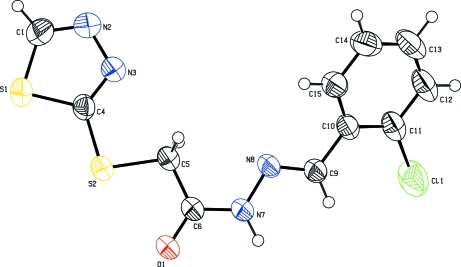
Perspective view of the molecule showing the thermal ellipsoids are drawn at 50% probability level.

**Fig. 2.**
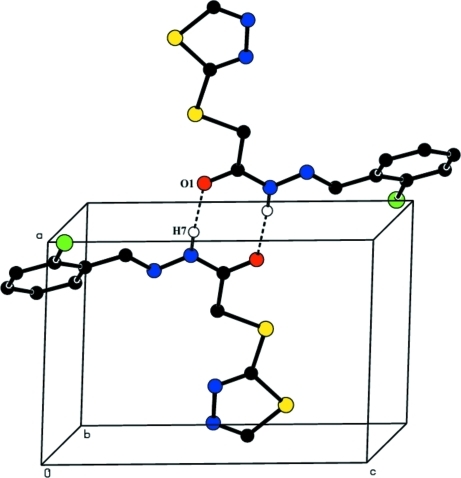
The crystal packing of the molecules viewed down b–axis. H atoms not involved in hydrogen bonding have been omitted for clarity.

## supplementary crystallographic information

### Comment

1,3,4-Thiadiazole derivatives are of interest because of their chemical and pharmaceutical properties. Some derivatives are useful in the preparation of intermediate for anticarcinogens. Recently many 1,3,4- thiadiazole nucleus have been synthesized and evaluated for their antiproliferative effect *in vitro* against the cells of various human tumor cell lines (Matysiak & Opolski, 2006). Some of the derivatives have effective antibacterial (Alireza *et al.*, 2005) and insecticidal activities (Wang *et al.*, 1999). In view of these facts and to ascertain the molecular conformation, crystallographic study of the title compound has been carried out.

The *ORTEP* plot of the molecule is shown in Fig.1. The thiadiazole and the chlorophenyl rings are planar and oriented at an angle of 43.1 (1)° with each other. The sum of the bond angles around the N7 atom (359.8°) of the acetohydrazide group in the molecule is in accordance with *sp^2^* hybridization. The packing of the molecules are controlled by N—H···O, C—H···Cl, C—H···π, π···π types of intra and intermolecular interactions. Atom N7 of the molecule at (*x*, *y*, *z*) donates a proton to atom O1 of the molecule at (2 - *x*, 1 - *y*, 1 - *z*) forming an intermolecular N—H···O bond which link the molecules into *R*_2_^2^(8) dimer (Bernstein *et al.*, 1995) as shown in Fig 2. The acetohydrazide group interacts with the thiadiazole ring moiety through a C—H···π interaction involving atom C5, the separation between H5A and the centroid of the S1/C1/N2/N3/C4 (*Cg*1) ring being 2.95 Å.

### Experimental

To a solution of 2-mercaptothiadiazole (50 g; 423 mmol) in acetone (500 ml) anhydrous sodium carbonate (24.66 g; 233 mmol) was added. Ethyl bromoacetate (70.65 g; 423 mmol) was added slowly to the reaction mixture at room temperature under stirring. The progress of the reaction was monitored by thin layer chromatography using ethyl acetate/n-hexane (3:7) as eluent. The bye-product sodium bromide was removed by filtration. The mother liquor was concentrated under vacuum to remove acetone and the residual acetone was removed by strip off with methanol to yield ethyl (1,3,4- thiadiazol-2-ylthio)acetate. The residue was dissolved in methanol (300 ml) and the clear solution hydrazine hydrate (42.35 g; 846 mmol) was added and heated under reflux. The progress of the reaction was monitored by thin layer chromatography using chloroform/methanol (9:1) as eluent. The reaction mass was cooled to 0–5° C. The crystallized product, 2-(1,3,4- thiadiazol-2-ylthio)acetohydrazide was filtered and washed with chilled methanol. To a mixture of isolated product (10 mmol) and 2-chlorobenzaldehyde (10 mmol) in ethanol (20 ml), a few drops of acetic acid was added. The reaction mixture was heated under reflux till completion of reaction. The reaction was monitored by thin layer chromatography using chloroform /methanol (8:2). The reaction mass was cooled to room temperature. The crystallized product, 2-[1,3,4-Thiadiazol-2-ylthio]-N'-[(1E) -(2-chlorophenyl)methylene]acetohydrazide was filtered and washed with ethanol.

### Refinement

The N bound H atom was refined and the C bound H atoms positioned geometrically (C—H=0.93–0.97 Å) and allowed to ride on their parent atoms, with 1.2 *U*_eq_(C) for all H atoms.

### Crystal data

**Table:**

C_11_H_9_ClN_4_OS_2_	*Z* = 2
*M**_r_* = 312.79	*F*(000) = 320
Triclinic, *P*1	*D*_x_ = 1.522 Mg m^−^^3^
Hall symbol: -P 1	Mo *K*α radiation, λ = 0.71073 Å
*a* = 7.551 (5) Å	Cell parameters from 1536 reflections
*b* = 8.743 (3) Å	θ = 1.9–28.6°
*c* = 11.269 (5) Å	µ = 0.58 mm^−^^1^
α = 69.295 (5)°	*T* = 293 K
β = 87.493 (4)°	Block, colorless
γ = 78.892 (5)°	0.20 × 0.17 × 0.16 mm
*V* = 682.6 (6) Å^3^	

### Data collection

**Table:**

Bruker SMART APEXII area-detector diffractometer	3446 independent reflections
Radiation source: fine-focus sealed tube	2858 reflections with *I* > 2σ(*I*)
graphite	*R*_int_ = 0.026
ω and φ scans	θ_max_ = 28.6°, θ_min_ = 1.9°
Absorption correction: multi-scan (*SADABS*; Bruker, 2008)	*h* = −10→10
*T*_min_ = 0.890, *T*_max_ = 0.911	*k* = −11→11
12836 measured reflections	*l* = −15→15

### Refinement

**Table:**

Refinement on *F*^2^	Primary atom site location: structure-invariant direct methods
Least-squares matrix: full	Secondary atom site location: difference Fourier map
*R*[*F*^2^ > 2σ(*F*^2^)] = 0.043	Hydrogen site location: inferred from neighbouring sites
*wR*(*F*^2^) = 0.121	H atoms treated by a mixture of independent and constrained refinement
*S* = 1.05	*w* = 1/[σ^2^(*F*_o_^2^) + (0.0552*P*)^2^ + 0.298*P*] where *P* = (*F*_o_^2^ + 2*F*_c_^2^)/3
3446 reflections	(Δ/σ)_max_ < 0.001
176 parameters	Δρ_max_ = 0.81 e Å^−^^3^
0 restraints	Δρ_min_ = −0.70 e Å^−^^3^

### Special details

**Table:**

Geometry. All e.s.d.'s (except the e.s.d. in the dihedral angle between two l.s. planes) are estimated using the full covariance matrix. The cell e.s.d.'s are taken into account individually in the estimation of e.s.d.'s in distances, angles and torsion angles; correlations between e.s.d.'s in cell parameters are only used when they are defined by crystal symmetry. An approximate (isotropic) treatment of cell e.s.d.'s is used for estimating e.s.d.'s involving l.s. planes.
Refinement. Refinement of *F*^2^ against ALL reflections. The weighted *R*-factor *wR* and goodness of fit *S* are based on *F*^2^, conventional *R*-factors *R* are based on *F*, with *F* set to zero for negative *F*^2^. The threshold expression of *F*^2^ > σ(*F*^2^) is used only for calculating *R*-factors(gt) *etc*. and is not relevant to the choice of reflections for refinement. *R*-factors based on *F*^2^ are statistically about twice as large as those based on *F*, and *R*- factors based on ALL data will be even larger.

### Fractional atomic coordinates and isotropic or equivalent isotropic displacement parameters (Å^2^)

**Table:**

	*x*	*y*	*z*	*U*_iso_*/*U*_eq_	
S1	0.25535 (9)	0.03479 (8)	0.74213 (5)	0.06663 (19)	
S2	0.56742 (7)	0.20903 (7)	0.65247 (4)	0.05462 (16)	
Cl1	0.84070 (13)	0.88994 (8)	−0.05412 (6)	0.0894 (3)	
O1	0.8486 (2)	0.3817 (2)	0.59757 (12)	0.0554 (3)	
C1	0.1207 (3)	0.0256 (3)	0.6276 (2)	0.0626 (5)	
H1	0.0209	−0.0258	0.6469	0.075*	
N2	0.1673 (3)	0.0946 (3)	0.51348 (18)	0.0629 (5)	
N3	0.3200 (3)	0.1628 (2)	0.50782 (16)	0.0562 (4)	
C4	0.3807 (3)	0.1407 (2)	0.61968 (17)	0.0463 (4)	
C5	0.6334 (3)	0.3041 (2)	0.49114 (16)	0.0471 (4)	
H5A	0.6700	0.2203	0.4524	0.057*	
H5B	0.5333	0.3857	0.4414	0.057*	
C6	0.7884 (2)	0.3867 (2)	0.49625 (16)	0.0438 (4)	
N7	0.8544 (2)	0.4699 (2)	0.38490 (14)	0.0489 (4)	
H7	0.950 (3)	0.519 (3)	0.386 (2)	0.065 (7)*	
N8	0.7873 (2)	0.4676 (2)	0.27372 (13)	0.0460 (3)	
C9	0.8396 (2)	0.5689 (2)	0.17253 (16)	0.0449 (4)	
H9	0.9113	0.6420	0.1773	0.054*	
C10	0.7866 (2)	0.5701 (2)	0.04874 (16)	0.0454 (4)	
C11	0.7840 (3)	0.7090 (3)	−0.06154 (18)	0.0556 (5)	
C12	0.7388 (3)	0.7078 (3)	−0.17847 (19)	0.0672 (6)	
H12	0.7370	0.8021	−0.2507	0.081*	
C13	0.6966 (3)	0.5678 (4)	−0.1873 (2)	0.0729 (7)	
H13	0.6667	0.5665	−0.2661	0.087*	
C14	0.6980 (3)	0.4283 (4)	−0.0810 (2)	0.0694 (6)	
H14	0.6692	0.3330	−0.0880	0.083*	
C15	0.7423 (3)	0.4294 (3)	0.0368 (2)	0.0562 (5)	
H15	0.7422	0.3348	0.1085	0.067*	

### Atomic displacement parameters (Å^2^)

**Table:**

	*U*^11^	*U*^22^	*U*^33^	*U*^12^	*U*^13^	*U*^23^
S1	0.0858 (4)	0.0739 (4)	0.0431 (3)	−0.0386 (3)	0.0118 (3)	−0.0131 (2)
S2	0.0671 (3)	0.0632 (3)	0.0327 (2)	−0.0252 (2)	−0.00281 (19)	−0.0087 (2)
Cl1	0.1404 (7)	0.0620 (4)	0.0558 (3)	−0.0234 (4)	−0.0051 (4)	−0.0055 (3)
O1	0.0617 (8)	0.0749 (9)	0.0336 (6)	−0.0256 (7)	−0.0014 (5)	−0.0173 (6)
C1	0.0675 (13)	0.0632 (13)	0.0646 (13)	−0.0273 (11)	0.0083 (10)	−0.0247 (10)
N2	0.0700 (11)	0.0703 (12)	0.0560 (10)	−0.0295 (9)	0.0011 (8)	−0.0228 (9)
N3	0.0681 (11)	0.0625 (10)	0.0417 (8)	−0.0259 (8)	0.0007 (7)	−0.0155 (7)
C4	0.0587 (11)	0.0410 (8)	0.0378 (8)	−0.0138 (8)	0.0045 (7)	−0.0102 (7)
C5	0.0586 (11)	0.0522 (10)	0.0329 (8)	−0.0178 (8)	0.0016 (7)	−0.0140 (7)
C6	0.0475 (9)	0.0489 (9)	0.0345 (8)	−0.0097 (7)	−0.0018 (7)	−0.0134 (7)
N7	0.0493 (9)	0.0647 (10)	0.0330 (7)	−0.0199 (8)	−0.0038 (6)	−0.0125 (7)
N8	0.0448 (8)	0.0608 (9)	0.0320 (7)	−0.0129 (7)	−0.0030 (6)	−0.0138 (6)
C9	0.0429 (9)	0.0537 (10)	0.0365 (8)	−0.0108 (7)	−0.0017 (7)	−0.0126 (7)
C10	0.0395 (8)	0.0597 (11)	0.0341 (8)	−0.0061 (7)	0.0009 (6)	−0.0148 (7)
C11	0.0578 (11)	0.0646 (12)	0.0369 (9)	−0.0040 (9)	−0.0001 (8)	−0.0127 (8)
C12	0.0677 (14)	0.0887 (17)	0.0342 (9)	−0.0003 (12)	−0.0031 (9)	−0.0154 (10)
C13	0.0602 (13)	0.118 (2)	0.0442 (11)	−0.0046 (13)	−0.0054 (9)	−0.0385 (13)
C14	0.0605 (13)	0.0982 (18)	0.0666 (14)	−0.0197 (12)	0.0004 (11)	−0.0470 (14)
C15	0.0530 (11)	0.0702 (13)	0.0489 (10)	−0.0155 (9)	0.0012 (8)	−0.0230 (9)

### Geometric parameters (Å, °)

**Table:**

S1—C1	1.711 (3)	N7—H7	0.91 (3)
S1—C4	1.725 (2)	N8—C9	1.273 (2)
S2—C4	1.734 (2)	C9—C10	1.464 (2)
S2—C5	1.8044 (19)	C9—H9	0.9300
Cl1—C11	1.747 (3)	C10—C15	1.387 (3)
O1—C6	1.231 (2)	C10—C11	1.394 (3)
C1—N2	1.278 (3)	C11—C12	1.380 (3)
C1—H1	0.9300	C12—C13	1.360 (4)
N2—N3	1.386 (3)	C12—H12	0.9300
N3—C4	1.296 (2)	C13—C14	1.373 (4)
C5—C6	1.502 (3)	C13—H13	0.9300
C5—H5A	0.9700	C14—C15	1.387 (3)
C5—H5B	0.9700	C14—H14	0.9300
C6—N7	1.338 (2)	C15—H15	0.9300
N7—N8	1.380 (2)		
			
C1—S1—C4	86.64 (11)	C9—N8—N7	115.02 (16)
C4—S2—C5	97.92 (9)	N8—C9—C10	119.93 (17)
N2—C1—S1	115.12 (18)	N8—C9—H9	120.0
N2—C1—H1	122.4	C10—C9—H9	120.0
S1—C1—H1	122.4	C15—C10—C11	117.46 (18)
C1—N2—N3	112.27 (18)	C15—C10—C9	120.55 (17)
C4—N3—N2	112.06 (17)	C11—C10—C9	121.96 (19)
N3—C4—S1	113.92 (16)	C12—C11—C10	121.7 (2)
N3—C4—S2	126.04 (15)	C12—C11—Cl1	118.25 (18)
S1—C4—S2	120.04 (11)	C10—C11—Cl1	120.05 (16)
C6—C5—S2	107.09 (12)	C13—C12—C11	119.5 (2)
C6—C5—H5A	110.3	C13—C12—H12	120.2
S2—C5—H5A	110.3	C11—C12—H12	120.2
C6—C5—H5B	110.3	C12—C13—C14	120.6 (2)
S2—C5—H5B	110.3	C12—C13—H13	119.7
H5A—C5—H5B	108.6	C14—C13—H13	119.7
O1—C6—N7	121.70 (17)	C13—C14—C15	120.0 (2)
O1—C6—C5	121.66 (16)	C13—C14—H14	120.0
N7—C6—C5	116.62 (15)	C15—C14—H14	120.0
C6—N7—N8	119.89 (16)	C10—C15—C14	120.7 (2)
C6—N7—H7	118.1 (16)	C10—C15—H15	119.6
N8—N7—H7	121.8 (16)	C14—C15—H15	119.6
			
C4—S1—C1—N2	−0.2 (2)	N7—N8—C9—C10	176.09 (16)
S1—C1—N2—N3	0.1 (3)	N8—C9—C10—C15	−24.7 (3)
C1—N2—N3—C4	0.1 (3)	N8—C9—C10—C11	157.42 (19)
N2—N3—C4—S1	−0.3 (2)	C15—C10—C11—C12	0.2 (3)
N2—N3—C4—S2	−179.38 (15)	C9—C10—C11—C12	178.18 (19)
C1—S1—C4—N3	0.26 (17)	C15—C10—C11—Cl1	−179.30 (15)
C1—S1—C4—S2	179.43 (14)	C9—C10—C11—Cl1	−1.3 (3)
C5—S2—C4—N3	−1.1 (2)	C10—C11—C12—C13	−0.5 (3)
C5—S2—C4—S1	179.81 (12)	Cl1—C11—C12—C13	179.03 (18)
C4—S2—C5—C6	175.22 (13)	C11—C12—C13—C14	0.3 (4)
S2—C5—C6—O1	0.3 (2)	C12—C13—C14—C15	0.1 (4)
S2—C5—C6—N7	−177.98 (14)	C11—C10—C15—C14	0.2 (3)
O1—C6—N7—N8	177.73 (17)	C9—C10—C15—C14	−177.78 (19)
C5—C6—N7—N8	−4.0 (3)	C13—C14—C15—C10	−0.4 (3)
C6—N7—N8—C9	170.33 (17)		

### Hydrogen-bond geometry (Å, °)

**Table:**

Cg1 is the centroid of the S1/C1/N2/N3/C4 ring.

**Table:**

*D*—H···*A*	*D*—H	H···*A*	*D*···*A*	*D*—H···*A*
C9—H9···Cl1	0.93	2.73	3.056 (2)	102.
N7—H7···O1^i^	0.91 (3)	1.93 (3)	2.845 (3)	175 (2)
C5—H5A···Cg1^ii^	0.97	2.95	3.896 (3)	165

Symmetry codes:  (i) −*x*+2, −*y*+1, −*z*+1; (ii) −*x*+1, −*y*, −*z*+1.
